# Co-generation of biohydrogen and biochemicals from co-digestion of *Chlorella* sp. biomass hydrolysate with sugarcane leaf hydrolysate in an integrated circular biorefinery concept

**DOI:** 10.1186/s13068-021-02041-6

**Published:** 2021-10-01

**Authors:** Napapat Sitthikitpanya, Sureewan Sittijunda, Sontaya Khamtib, Alissara Reungsang

**Affiliations:** 1 Department of Biotechnology, Faculty of Technology, Khon Kaen University, Khon Kaen, Thailand; 2 Faculty of Environment and Resource Studies, Mahidol Univesity, Nakhon Pathom, Thailand; 3 Soil Science Research Group, Agricultural Production Science Research and Development Division, Department of Agriculture, Bangkok, Thailand; 4 Research Group for Development of Microbial Hydrogen Production Process from Biomass, Khon Kaen University, Khon Kaen, Thailand; 5 Academy of Science, Royal Society of Thailand, Bangkok, Thailand

**Keywords:** Anaerobic digestion, Polyhydroxyalkanoates (PHAs), Lipid, Soil supplement, Microalgae

## Abstract

**Background:**

A platform for the utilization of the *Chlorella* sp. biomass and sugarcane leaves to produce multiple products (biorefinery concept) including hydrogen, methane, polyhydroxyalkanoates (PHAs), lipid, and soil supplement with the goal to achieve the zero waste generation (circular economy) is demonstrated in this study. Microalgal biomass were hydrolyzed by mixed enzymes while sugarcane leaves were pretreated with alkali followed by enzyme. Hydrolysates were used to produce hydrogen and the hydrogenic effluent was used to produce multi-products. Solid residues at the end of hydrogen fermentation and the remaining acidified slurries from methane production were evaluated for the compost properties.

**Results:**

The maximum hydrogen yield of 207.65 mL-H_2_/g-volatile solid (VS)_added_ was obtained from 0.92, 15.27, and 3.82 g-VS/L of *Chlorella* sp. biomass hydrolysate, sugarcane leaf hydrolysate, and anaerobic sludge, respectively. Hydrogenic effluent produced 321.1 mL/g-VS of methane yield, 2.01 g/L PHAs concentration, and 0.20 g/L of lipid concentration. Solid residues and the acidified slurries at the end of the hydrogen and methane production process were proved to have compost properties.

**Conclusion:**

Hydrogen production followed by methane, PHA and lipid productions is a successful integrated circular biorefinery platform to efficiently utilize the hydrolysates of *Chlorella* sp. biomass and sugarcane leaf. The potential use of the solid residues at the end of hydrogen fermentation and the remaining acidified slurries from methane production as soil supplements demonstrates the zero waste concept. The approach revealed in this study provides a foundation for the optimal use of feedstock, resulting in zero waste.

**Graphic Abstract:**

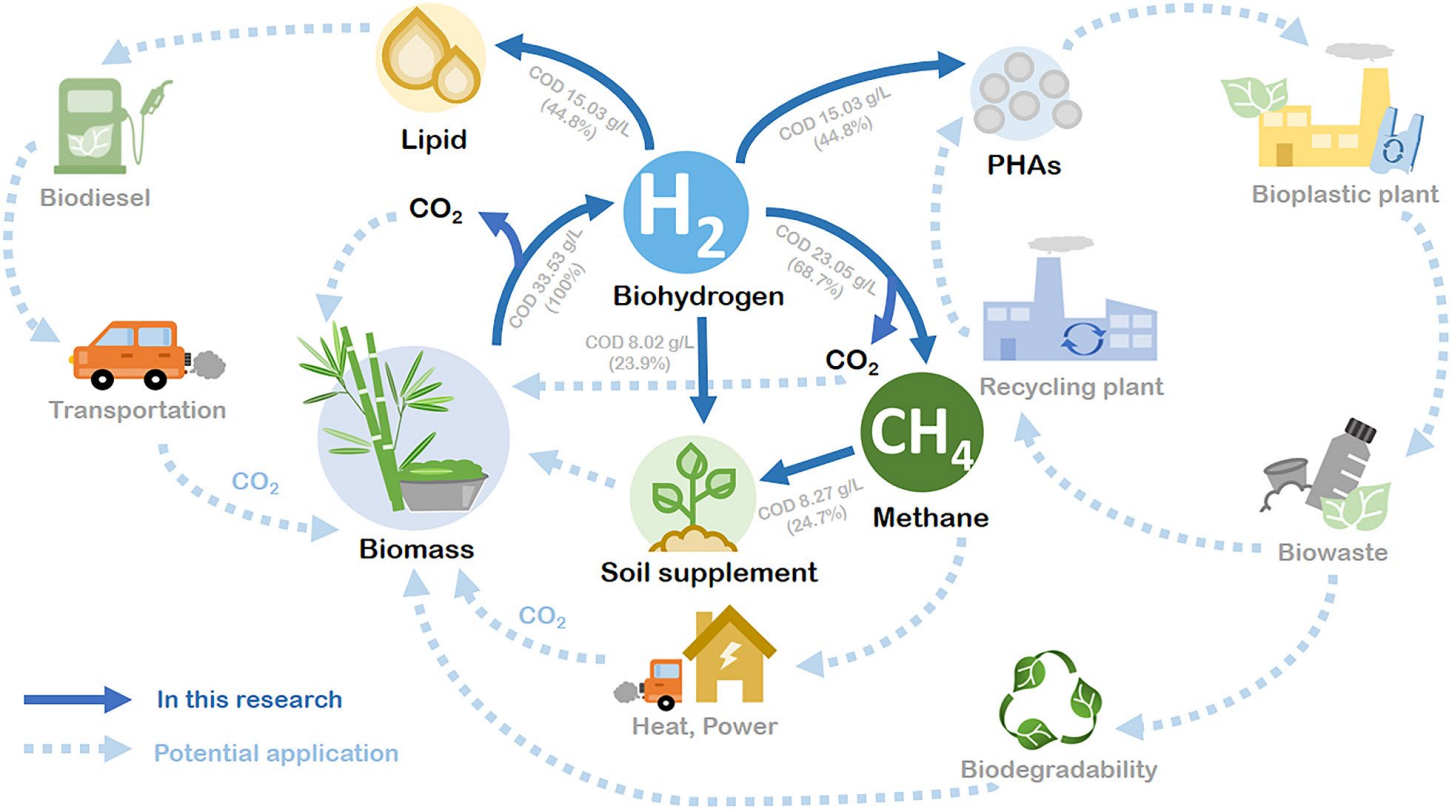

**Supplementary Information:**

The online version contains supplementary material available at 10.1186/s13068-021-02041-6.

## Background

Biohydrogen is a renewable energy resource that has received attention because of its sustainability, clean combustion product, and high energy content of 122 kJ/g, which is 2.5 times that of fossil fuels [1]. Biohydrogen is widely produced by dark fermentation due to ease of operation and low operation cost, a high production rate, and commercialization potential [2]. Recently, microalgal biomass has been used to produce biohydrogen by dark fermentation [3]. Microalgae has high protein and carbohydrate content and low lignin levels, However, the low carbon-to-nitrogen (C/N) ratio causes ammonia inhibition [4, 5], resulting in low hydrogen productivity. Thus, co-digestion with a carbon-rich substrate under a suitable C/N ratio is required to eliminate this drawback. Co-digestion involves the use of mixtures of substrates. Its advantages include dilution of toxic compounds, adjustment of the C/N ratio, improved nutrient balance, enhanced synergistic reactions of microorganisms, and increased fermentation efficiency [5, 6]. In the present study, microalgal biomass was co-digested with sugarcane leaves with high carbon content to attain the proper C/N ratio for the production of hydrogen.

After dark fermentation, a large amount of the effluent, also known as hydrogenic effluent, containing high concentrations of volatile fatty acids (VFAs), such as acetic, butyric, and propionic acids, is generated. The chemical oxygen demand (COD) of the hydrogenic effluent has a high COD of approximately 20,000–35,000 mg/L [7, 8], which can cause environmental problems upon discharge. Previous studies have reported the use of hydrogenic effluents to produce bioenergy and biochemicals. For example, a methane yield (MY) of 10.77 mL/g-COD under an optimal hydraulic retention time (HRT) of 9 d was obtained from the hydrogenic effluent of a co-digestion of swine manure and pineapple waste process [9]. The effluent discharged after biohydrogen production from the co-digestion of microalgae with organic wastes, including molasses, Napier grass (*Pennisetum purpureum*), empty fruit bunches (EFB), palm oil mill effluent (POME), and glycerol waste gave the highest MY of 214–577 mL/g-VS [5]. Hou et al. [10] found that methane production (14.96 kJ/g-VS_added_) from hydrogenic effluent of food waste supplemented with air-nanobubble water resulted in a high total energy yield (EY) of 15.31 kJ/g-VS_added_, which is 43.7 times greater than that of hydrogen production (0.35 kJ/g-VS_added_). These results demonstrate that methane production from hydrogenic effluent significantly improved the total EY. Thus, the two-stage hydrogen and methane production process is a promising process for recovering energy from biomass. In addition to methane, polyhydroxyalkanoates (PHAs) are produced from hydrogenic effluent. Colombo et al. [11] reported that the hydrogenic effluent from the fermentation of second cheese whey (SCW) and concentrated cheese whey permeate (CCWP) was bio-converted to PHAs, resulting in a maximum PHA yield of 0.77 ± 0.14 and 0.72 ± 0.14 mg-COD_PHA_/mg-COD_organic acid-in_, respectively. Zhao et al. [12] found that PHA accumulation of 15.8% of volatile suspended solids (VSS) was achieved from the hydrogenic effluent of original hydrolyzed polyacrylamide-containing wastewater at a C/N ratio of 51. Moreover, PHAs of 40% of the dry cell weight (DCW) were obtained from the effluent obtained after biohydrogen production from distillery waste [13]. These studies indicate the efficacy of VFAs in the production of PHAs. The synthesis of lipids from the hydrogenic effluent has also been studied. Ren et al. [14] found that lipid production from the hydrogenic effluent of stimulated food waste produced the highest lipid production (515.6 mg/L) by *Scenedesmus* sp. R-16. Mu et al. [15] showed that after the dark fermentation of pretreated duckweed with 1% H_2_SO_4_, the hydrogenic effluent was used to cultivate *Chlorella saccharophila* FACHB-4, resulting in lipid concentrations and contents of 63.4–270.9 mg/L and 12.0–37.4%, respectively. The production of hydrogen and lipids from starch wastewater by co-culture of oleaginous microalgae with anaerobic sludge was studied by Ren et al. [16]. A maximum total lipid concentration of 0.36 g/L was observed at a starch concentration of 6 g/L, optimal ratio of 30:1, and initial pH of 8. These results indicate the successful utilization of hydrogenic effluent for lipid production by microalgae. However, in these studies of hydrogenic effluent from various types of biomass, only one product, (methane, PHA, or lipid) was obtained. To the best of our knowledge, however, our study is the first to report the use of hydrogenic effluent from *Chlorella* sp. biomass co-digested with sugarcane leaves to produce multiple products without generating waste (the so-called integrated circular biorefinery concept).

Therefore, the aim of our study was to develop a platform for the utilization of the biomass of *Chlorella* sp. and sugarcane leaves to produce multiple products (biorefinery concept) including bioenergy, i.e., hydrogen, and methane, and biochemicals, i.e., PHAs, lipid, and soil supplement with the goal to achieve the zero waste generation (circular economy).

## Results and discussion

### Feedstock compositions and sugarcane leaves pretreatments

Analysis of *Chlorella* sp. biomass and its hydrolysate, sugarcane leaf and its hydrolysate, and anaerobic sludge compositions were conducted. The *Chlorella* sp. biomass consists of (all in % (w/w)): protein 52.8; carbohydrate 29.2; lipid 8.7; and ash 5.0 [17]. The sugarcane leaves are composed of (all in % (w/w)): cellulose 36.69, hemicellulose 17.49, lignin 10.35, and ash 12.62. The main composition found in both *Chlorella* sp. biomass hydrolysate and sugarcane leaf hydrolysate was carbon, followed by nitrogen and hydrogen (Additional file 1: Table S1). The C/N ratio of the sugarcane leaf hydrolysate was 15.52 and 14.83 times higher than the hydrolysates obtained from *Chlorella* sp. biomass and anaerobic sludge, respectively. As a result, sugarcane leaf hydrolysate is an excellent choice for balancing the nutrients in the fermentation medium.

**Fig. 1:**
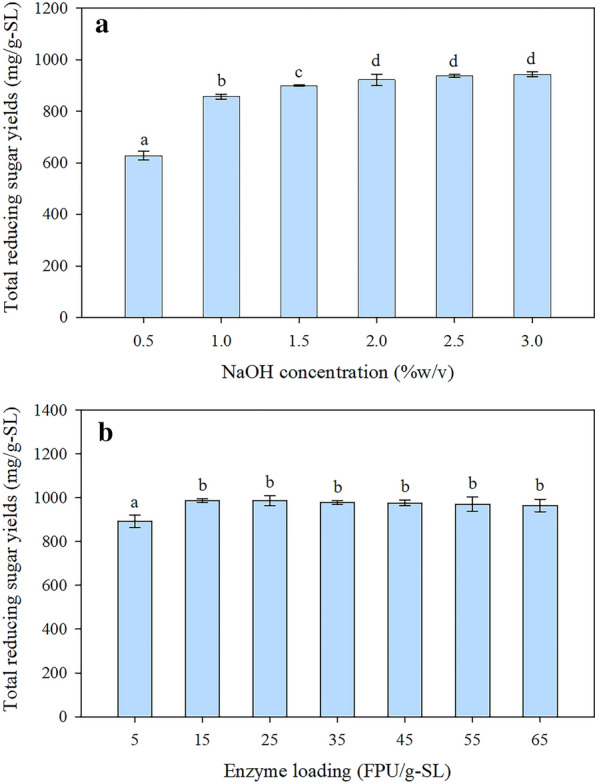
The TRS yield of sugarcane leaves after alkaline pretreatment and enzymatic hydrolysis. **a** The TRS yield of sugarcane leaf hydrolysate at different NaOH concentrations. The sample (10%, w/v) was pretreated with NaOH in an autoclave at 121 °C for 60 min, then hydrolyzed using Cellic^®^ CTec2 (35 FPU/g-sugarcane leaves) at 50 °C and 150 rpm for 72 h. **b** The TRS yield from enzymatic hydrolysis of sugarcane leaves pretreated with 2% (w/v) NaOH at different enzyme loadings. Same-alphabet values are not significantly different (ρ < 0.05). Data are mean triple experiments ± SD

Pretreatment conditions of sugarcane leaves by sodium hydroxide (NaOH) and enzyme were optimized to achieve a high sugar yield. The total reducing sugar (TRS) yield increased with increasing NaOH concentration from 0 to 2% (w/v) (Fig. 1). Nevertheless, the TRS yield increased slightly when the NaOH concentration was greater than 2% (w/v) (Fig. 1a). The results suggest that alkaline pretreatment improved the efficiency of cellulose degradation for hydrolysis by enzymes. Generally, alkaline is used to remove lignin from the lignocellulosic biomass structure. The delignification process significantly affects the solubility of hemicellulose [18]. The reaction mechanism is based on the saponification of intermolecular ester bonds, crosslinking lignin and hemicellulose [19]. Saponification allows the breakdown of lignin–carbohydrate complex (LCC) connections and gives the cellulose greater access to the enzyme during hydrolysis [18, 19]. Moreover, pretreatment with NaOH also led to swelling of the cellulose polymeric chain. Consequently, the surface area and accessibility of enzymes to the biomass are increased [20]. The highest TRS yield of 942.8 mg/g-sugarcane leaves was obtained when sugarcane leaves were pretreated with 3% (w/v) NaOH. However, the TRS yield was not significantly different when compared with the TRS yield at NaOH concentrations of 2, 2.5, and 3% (w/v). Thus, a 2% (w/v) NaOH was chosen as the optimal dosage to pretreat the sugarcane leaves. Using low alkaline concentrations would decrease operating costs, chemicals, and waste.

The TRS yield from enzymatic hydrolysis of sugarcane leaves pretreated with the optimum NaOH concentration (2% (w/v)) is depicted in Fig. 1b. Increasing the enzyme loading from 5 to 15 filter paper unit (FPU)/g-sugarcane leaves resulted in a noticeable increase in the TRS yield from 891.2 to 985.5 mg/g-sugarcane leaves. However, the TRS yields did not significantly increase when the enzyme loading was greater than 15 FPU/g-sugarcane leaves (Fig. 1b). The results demonstrate that an enzyme loading higher than 15 FPU/g-sugarcane leaves is not necessary for the hydrolysis reaction. At high enzyme loading (> 15 FPU/g-sugarcane leaves), the adsorption efficiency of the enzyme is reduced by saturation of the enzyme on the cellulose surface [21]. The results indicate that the use of enzyme loading at 15 FPU/g-sugarcane leaves was sufficient to degrade the cellulose structure. Moreover, a low loading of enzymes is preferable over high loading because it lowers the operational costs [2, 21]. Thus, the optimum conditions for alkaline pretreatment and enzymatic hydrolysis of sugarcane leaves were 2% (w/v) NaOH concentration and 15 FPU/g-sugarcane leaves. Under these conditions, a TRS yield of 985.5 mg/g-sugarcane leaves was achieved, which was 14.1 times higher than that of untreated sugarcane leaves.

### Biohydrogen production from a co-digestion of *Chlorella* sp. biomass hydrolysate and sugarcane leaf hydrolysate

Factors affecting the co-digestion of hydrolysates of *Chlorella* sp. biomass and sugarcane leaf with anaerobic sludge to produce hydrogen were optimized. D-optimal mixture design was used to design the experiments. The biohydrogen production from the co-digestion of *Chlorella* sp. biomass hydrolysate (X_1_), sugarcane leaf hydrolysate (X_2_), and anaerobic sludge (X_3_) is presented in Table 1. Regression analysis of the experimental data (Table 1) resulted in the following cubic equation for predicting hydrogen production potential (P_s_) (Eq. (1)):

1 $${Y_{{{\rm{P}}_{\rm{s}}}}} = 82.49{\rm{X}_1} + 113.18{\rm{X}_2} + 1.05{\rm{X}_3} - 4.66{\rm{X}_1}{\rm{X}_2} - 5.19{{\rm{X}}_1}{{\rm{X}}_3} + 13.36{{\rm{X}}_2}{{\rm{X}}_3} + 2.80{{\rm{X}}_1}{{\rm{X}}_2}{{\rm{X}}_3} - 0.37{{\rm{X}}_1}{{\rm{X}}_2}\left( {{{\rm{X}}_1} - {{\rm{X}}_2}} \right) + 0.83{{\rm{X}}_1}{{\rm{X}}_3}\left( {{{\rm{X}}_1} - {{\rm{X}}_3}} \right) + 1.85{{\rm{X}}_2}{{\rm{X}}_3}\left( {{{\rm{X}}_2} - {{\rm{X}}_3}} \right).$$

**Table 1:** Mixture design defining the proportions of *Chlorella* sp. biomass hydrolysate, sugarcane leaf hydrolysate, anaerobic sludge, and hydrogen production potential results

Run	*Chlorella* sp. biomass hydrolysate (g-VS/L)	Sugarcane leaf hydrolysate (g-VS/L)	Anaerobic sludge (g-VS/L)	*P*_s_ (mL-H_2_/L_substrate_)	
				Observed	Predicted
1	6.67	6.67	6.67	2239	2298
2	0.00	20.00	0.00	2032	2264
3	0.00	10.00	10.00	2454	2478
4	16.61	3.39	0.00	1219	1218
5	0.00	10.00	10.00	2455	2478
6	0.00	20.00	0.00	2471	2264
7	3.39	3.35	13.26	364	216
8	3.35	13.33	3.31	3660	3513
9	20.00	0.00	0.00	1630	1650
10	10.00	0.00	10.00	216	316
11	13.27	3.36	3.37	1964	1818
12	0.00	0.00	20.00	8	21
13	0.00	0.00	20.00	10	21
14	10.00	10.00	0.00	1442	1491
15	6.57	6.79	6.64	2264	2329
16	20.00	0.00	0.00	1646	1650
17	10.00	0.00	10.00	366	316
18	6.67	6.67	6.67	2267	2298
19	6.67	6.67	6.67	2230	2298

The model was significant (*ρ* < 0.05) at the 95% confidence level. The coefficient of determination (*R*^2^) of 0.9897 and adjusted *R*^2^ of 0.9795 indicate that the model can explain 97–99% of the variability of the response variable (Additional file 1: Table S2). Additionally, the lack of fit of the model is not significant (*ρ* = 0.1367), indicating that the model is valid and properly describes the *P*_s._

A three-dimensional (3D) response surface plot based on Eq. (1) was created to determine the interaction effects of the three factors on *P*_s_ (Additional file 1: Fig. S1). The optimum proportions of the feedstock were the hydrolysates of *Chlorella* sp. biomass and sugarcane leaf, and anaerobic sludge of 0.92, 15.27, and 3.82 g-VS/L, respectively. Under these proportions, the maximum P_s_ of 3,953 mL-H_2_/L_substrate_ was predicted. At these proportions, the C/N ratio was 20.08, which falls within a suitable range of C/N (20-30) for hydrogen production [22]. The confirmation experiment was conducted under the optimal conditions to verify the model. The *P*_s_ of 4,153 mL-H_2_/L_substrate_ was observed under optimum conditions, which is only 5.1% different from the predicted *P*_s_. The results suggested that the cubic model was valid for optimizing the feedstock proportions for hydrogen production. At the optimum condition, the observed *P*_s_ (4,153 mL-H_2_/L_substrate_) was 1.5 and 0.8 times higher than mono-digestion of *Chlorella* sp. biomass hydrolysate and sugarcane leaf hydrolysate alone. In addition, it was 414.4 times higher than the mono-digestion of anaerobic sludge. The results demonstrated that the co-digestion process under suitable proportions provided an appropriate C/N ratio and nutrient balance inside the hydrogen fermentation system. A proper C/N ratio of the substrate is essential for the growth and activity of fermentative microorganisms [23]. The excess C/N ratio inhibits the growth and metabolism of microorganisms, resulting in ineffective substrate utilization and hydrogen production. In contrast, a low C/N ratio indicates that the substrate has an excess nitrogen source, which may cause ammonia inhibition and is not suitable for hydrogen production [24].

The results from this study were compared to the literature search which were conducted on a co-digestion of microalgae with various kinds of biomass. We found that the hydrogen yield (HY) obtained under the optimum conditions in this study (207.65 mL-H_2_/g-VS_added_) was higher than that of the pretreated rice residue co-digested with microalgae (*Chlorella pyrenoidosa*) at a C/N ratio of 17.61 (201.8 mL-H_2_/g-VS) [25], and the macroalgae (*Laminaria digitata*) co-digested with microalgae (*Arthrospira platensis*) at a C/N ratio of 26.2 (85.0 mL-H_2_/g-VS) [3]. However, at the same C/N ratio of 20, the HY in this study was 3.75 times greater than HY from the co-digestion of *A. platensis* and *L. digitata* (55.3 mL-H_2_/g-VS) [26]. Results implied that C/N ratios as well as the types of biomass used in the co-digestion process affect the efficiency of hydrogen production.

The synergistic and antagonistic effects of the co-digestion process were investigated by dividing experimental *P*_s_ by calculated *P*_s_ (Table 2). The synergistic effect and the antagonistic effect of mixture proportions are indicated by the ratio higher and lower than 1, respectively, while the ratio is equal to 1 indicates that the substrate work independently from the mixture. The experimental P_s_ was obtained from volume of hydrogen produced by each proportion from experimental design while the calculated P_s_ was obtained from the P_s_ based on the VS of each substrate contained in the mixture [6]. An antagonistic effect was found in Runs 2, 4, 7, 10, 12, 14, and 17, while Runs 9 and 16 indicated that the substrate works independently from the mixture (Table 2). In contrast, other experimental runs showed a synergistic effect. At the optimal proportions, an experimental P_s_/calculated P_s_ ratio of 2.32 was achieved, indicating a synergistic effect. The results demonstrate that co-digestion at a suitable proportion promotes the interaction between the feedstock and microorganisms, which evidenced by a high P_s_. Moreover, synergism in co-digestion may be due to the contribution of enzymes, nutrients, additional trace elements, or any other amendment caused by the co-substrates, resulting in improved biological degradation of substrates and hydrogen production.

**Table 2:** Synergistic and antagonistic effects of co-digestion proportions on hydrogen production potential

Run	Experimental *P*_s_ (mL-H_2_/L_substrate_)	Calculated *P*_s_ (mL-H_2_/L_substrate_)	Experimental *P*_s_/calculated *P*_s_ ratio^*^
1	2239	1299	1.72
2	2032	2251	0.90
3	2454	1130	2.17
4	1219	1742	0.70
5	2455	1130	2.17
6	2471	2251	1.10
7	364	661	0.55
8	3661	1777	2.06
9	1630	1638	1.00
10	216	823	0.26
11	1964	1466	1.34
12	9	9	0.93
13	10	9	1.07
14	1442	1945	0.74
15	2264	1305	1.73
16	1646	1638	1.00
17	366	823	0.44
18	2267	1299	1.74
19	2230	1299	1.72
Optimum proportions	3953	1791	2.32

^*^Experimental *P*_s_/calculated *P*_s_ ratio < 1; antagonistic effect

^*^Experimental *P*_s_/calculated *P*_s_ ratio = 1; the substrate work independently from the mixture

^*^Experimental *P*_s_/calculated *P*_s_ ratio > 1; synergistic effect

### Methane production from hydrogenic effluent and energy recovery

The hydrogenic effluent in all experimental runs were further used as a substrate to produce methane in the second stage. Using the hydrogenic effluent under the optimum proportions to produce methane gave the MY of 321.1 mL/g-VS (equivalent to 13.8 kJ/g-VS) (Table 3). The EY under optimum conditions was 6 and 1.2 times, respectively, higher than that of the hydrogen and methane production processes. The results revealed that the two-stage hydrogen and methane production process significantly improved the energy recovery.

**Table 3:** Biogas and energy yield (EY) in each run of two-stage hydrogen and methane productions

Run	Yield (mL/g-VS)			EY (kJ/g-VS)		
	H_2_	CH_4_	Total	H_2_	CH_4_	Total
1	112.0	241.6	353.6	1.2	8.7	9.9
2	101.6	346.7	448.3	1.1	12.4	13.5
3	122.7	254.4	377.1	1.3	9.1	10.5
4	60.9	262.7	323.6	0.7	9.4	10.1
5	122.7	258.9	381.6	1.3	9.3	10.6
6	123.5	329.6	453.1	1.3	11.8	13.2
7	18.2	199.2	217.4	0.2	7.1	7.3
8	183	327.3	510.3	2.0	11.7	13.7
9	81.5	276.1	357.6	0.9	9.9	10.8
10	10.8	225.0	235.8	0.1	8.1	8.2
11	98.2	290.4	388.6	1.1	10.4	11.5
12	0.4	115.3	115.7	0.0	4.1	4.1
13	0.5	127.1	127.6	0.0	4.6	4.6
14	72.1	312.3	384.4	0.8	11.2	12.0
15	113.2	259.7	372.9	1.2	9.3	10.5
16	82.3	271.8	354.1	0.9	9.7	10.6
17	18.3	194.4	212.7	0.2	7.0	7.2
18	113.3	252.4	365.7	1.2	9.1	10.3
19	111.5	276.7	388.2	1.2	9.9	11.1
Optimum proportions	207.7	321.1	528.8	2.3	11.5	13.8

**Fig. 2:**
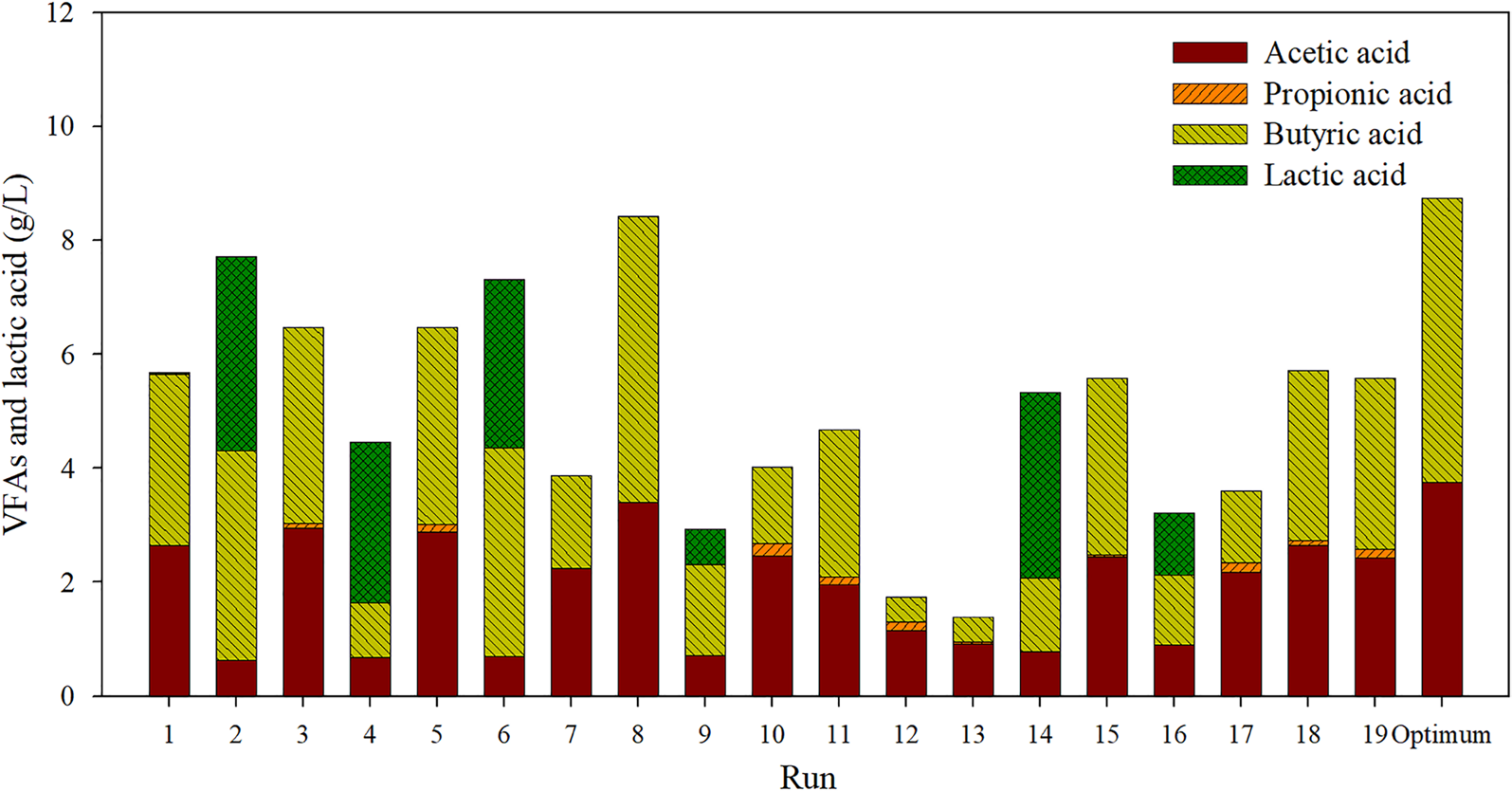
Volatile fatty acids (VFAs) and lactic acid profiles of hydrogenic effluent in each experimental run

The profiles of VFAs and lactic acid of hydrogenic effluent in each experimental run are depicted in Fig. 2. Acetic acid and butyric acid were the primary soluble metabolite products (SMPs) found in all experimental runs, whereas a small amount of propionic acid and lactic acid were found in some experimental runs. Results implied that acetic acid and butyric acid were the primary VFAs being bio-converted into methane by anaerobic mixed cultures in the second stage.

The comparison of HY, MY, and EY of this study with the literature search is shown in Table 4. The HY obtained in this study was relatively high compared to other studies, whereas MY was comparable. Consequently, the overall EY obtained in this study was higher than that reported in other studies [4, 26, 27]. The differences in HY and MY were due to the types of substrate, inoculum, and fermentation conditions. Fermentation conditions, especially pH, temperature, and nutrients, affected the production yield. Therefore, key factors affecting hydrogen and methane production must be optimized to enhance the production yield.

**Table 4:** Comparing hydrogen, methane, and energy yield of this study with the related literature search

Substrate	C/N ratio	H_2_ yield (mL-H_2_/g-VS)	CH_4_ yield (mL-CH_4_/g-VS)	EY (kJ/g-VS)	References
Food waste + nanobubble water supplementation	N/A	27.3	373.6	15.3^a^	[10]
Corn stover	N/A	69.0	249.4	9.7^b^	[27]
Macroalgae (*Laminaria* *digitata*) + Microalgae (*Arthrospira platensis*)	20.0	55.3	245.0	7.3^a^	[26]
Food waste + sewage sludge + 1% glycerol	N/A	140.2	342.0	15.5^a^	[53]
Food waste + sewage sludge + 3% glycerol	N/A	177.0	224.4	11.2^a^	
Macroalgae (*L. digitata*) + Microalgae (*Chlorella pyrenoidosa*)	20.0	97.0	224.3	9.1^b^	[4]
Macroalgae (*L. digitata*) + Microalgae (*Nannochloropsis oceanica*)	20.0	94.5	295.9	11.6^b^	
*Chlorella* sp. biomass hydrolysate + sugarcane leaf hydrolysate + anaerobic sludge	20.1	207.7	321.1	13.8^b^	This study

*H*_*2*_* yield* hydrogen yield (mL-H_2_/g-VS), *CH*_*4*_* yield* methane yield (mL-CH_4_/g-VS), *EY* energy yield (kJ/g-VS), *C/N ratio* carbon-to-nitrogen ratio, *VS* volatile solid, *N/A *not available

^a^Reported value

^b^Calculated value

### PHA production

**Fig. 3:**
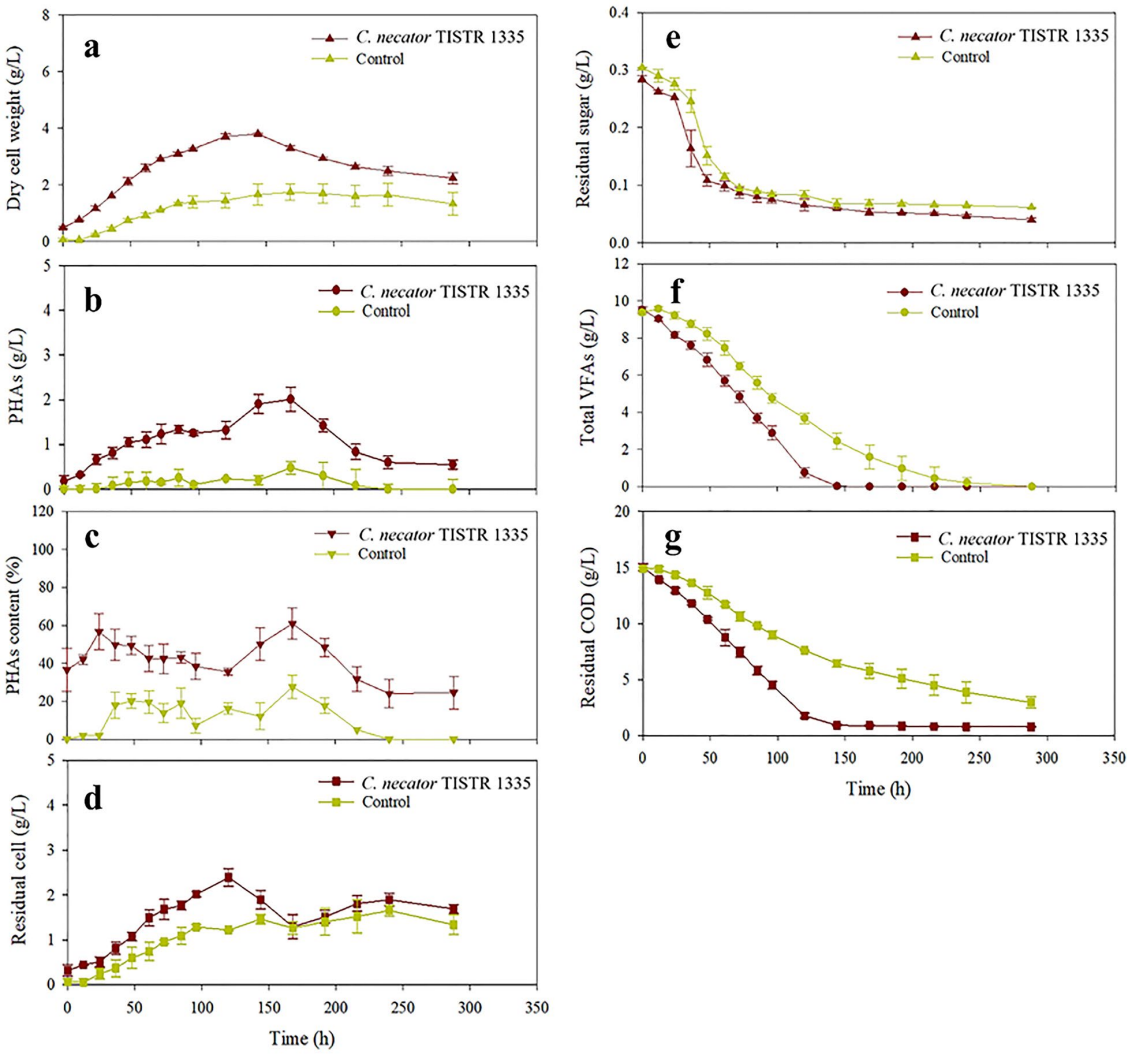
The time profile of cell growth and PHA production. **a** DCW, **b** PHA concentration, **c** PHA content, **d** residual cell concentration, **e** residual sugars concentration, **f** total volatile fatty acids (VFAs) concentration, **g** residue COD concentration. The PHAs was produced by fermenting hydrogenic effluent with *C. necator* at 30 °C and 200 rpm for 12 days

The hydrogenic effluent from the optimum proportions of feedstock that gave the highest P_s_ was further used for PHA production by *Cupriavidus necator*. In the treatment with *C. necator*, the biomass concentration rapidly increased and reached a maximum value of 3.80 g/L at 144 h and subsequently decreased over time (Fig. 3a). The PHA concentration rose steadily to the highest value of 2.01 g/L at 168 h and then dropped (Fig. 3b). The decrease in biomass and PHA concentrations could be due to the PHAs stored in bacterial cells being degraded to generate energy for cell maintenance and growth [28]. A maximum PHA content of 60.9% (w/w) and PHA yield of 0.14 g/g-COD_consumed_ were achieved at 168 h in the inoculation treatment (Fig. 3c). The PHA concentration, content, and yield of 0.48 g/L, 27.6% (w/w), and 0.05 g/g-COD_consumed_ were obtained for the control experiment (without the inoculum). The profiles of residual cell concentrations were correlated with the substrate utilization profiles. In the inoculated treatment, residual cell concentration increased rapidly to a maximum of 2.39 g/L at 120 h and then decreased gradually (Fig. 3d). The decrease in residual cell concentration indicated that the feedstock was consumed for PHA production rather than biomass production. The results showed that the VFAs in the hydrogenic effluent could be effectively used to produce PHAs by *C. necator*.

Residual sugar concentration in the treatments with and without *C. necator* was immediately decreased from around 0.3–0.1 g/L within 61 h and gradually decreased to a concentration below 0.1 g/L at the end of fermentation time (Fig. 3e). VFAs were simultaneously consumed with residual sugar, in which total VFAs were completely depleted after 144 and 288 h for inoculum and without inoculum treatment, respectively (Fig. 3f). The results demonstrated that *C. necator* and indigenous microorganisms in the hydrogenic effluent could consume residual sugars and VFAs for biomass and PHA production.

The COD concentration profiles (Fig. 3g) were similar to the total VFAs concentration profiles (Fig. 3f) for both treatments. In the treatment with *C. necator*, the COD concentration continuously decreased during 144 h and remained relatively stable thereafter, whereas the COD concentration in the control treatment decreased steadily over 288 h (Fig. 3g). The COD was virtually eliminated with removal percentages of approximately 95 and 80% for treatment with and without *C. necator*, respectively. The results demonstrate that the integration production process could convert 97.7% of the COD into hydrogen and PHAs.

**Fig. 4:**
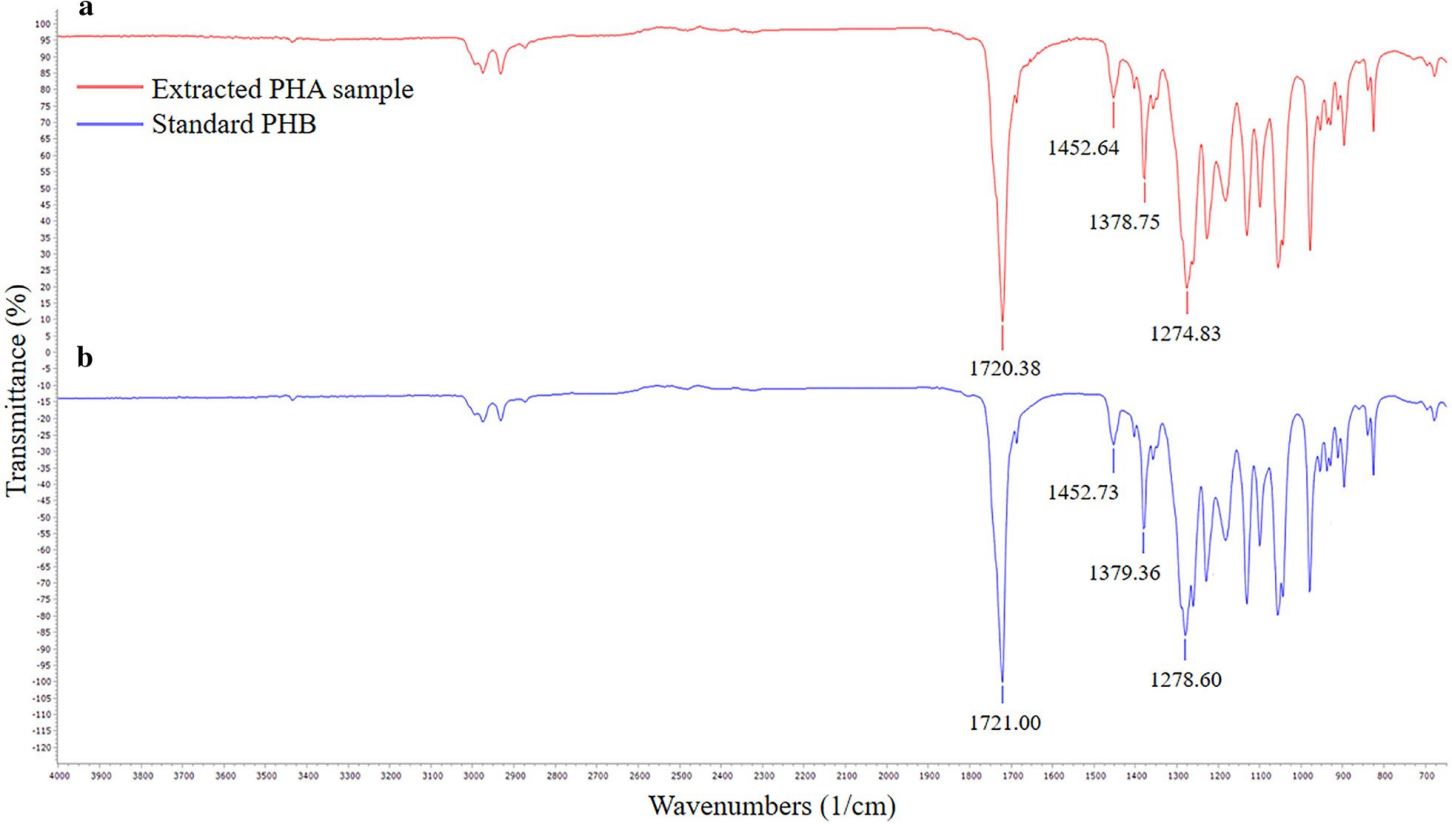
The FTIR spectrum. **a** Extracted PHA sample from *C. necator* grown on hydrogenic effluent, **b** standard PHB

The extracted PHA samples were verified using Fourier transform infrared spectrometer (FTIR). The samples showed peaks at wave numbers of 1720.38, 1378.75, 1452.64, and 1274.83 cm^−1^, corresponding to the C=O bond carbonyl groups, –CH_3_, –CH_2_, and –CH groups, respectively (Fig. 4a). These peaks correspond to peaks obtained at 1721.00, 1379.36, 1452.73, and 1278.60 cm^−1^ for standard polyhydroxybutyrate (PHB) (Sigma Aldrich, USA), confirming that the extracted polymer was PHB (Fig. 4b).

In comparison to other literature search, we found that PHA content of this study (60.9% w/w) was equivalent to PHA content produced from the hydrogenic effluent of thermal-hydrolyzed sludge (61.4% w/w) [29] and the hydrogenic effluent of second cheese whey (62.0% w/w) [11]. Results demonstrated that hydrogenic effluent from *Chlorella* sp. biomass co-digested with sugarcane leaves is a potential feedstock for PHA production.

### Lipid production

**Fig. 5:**
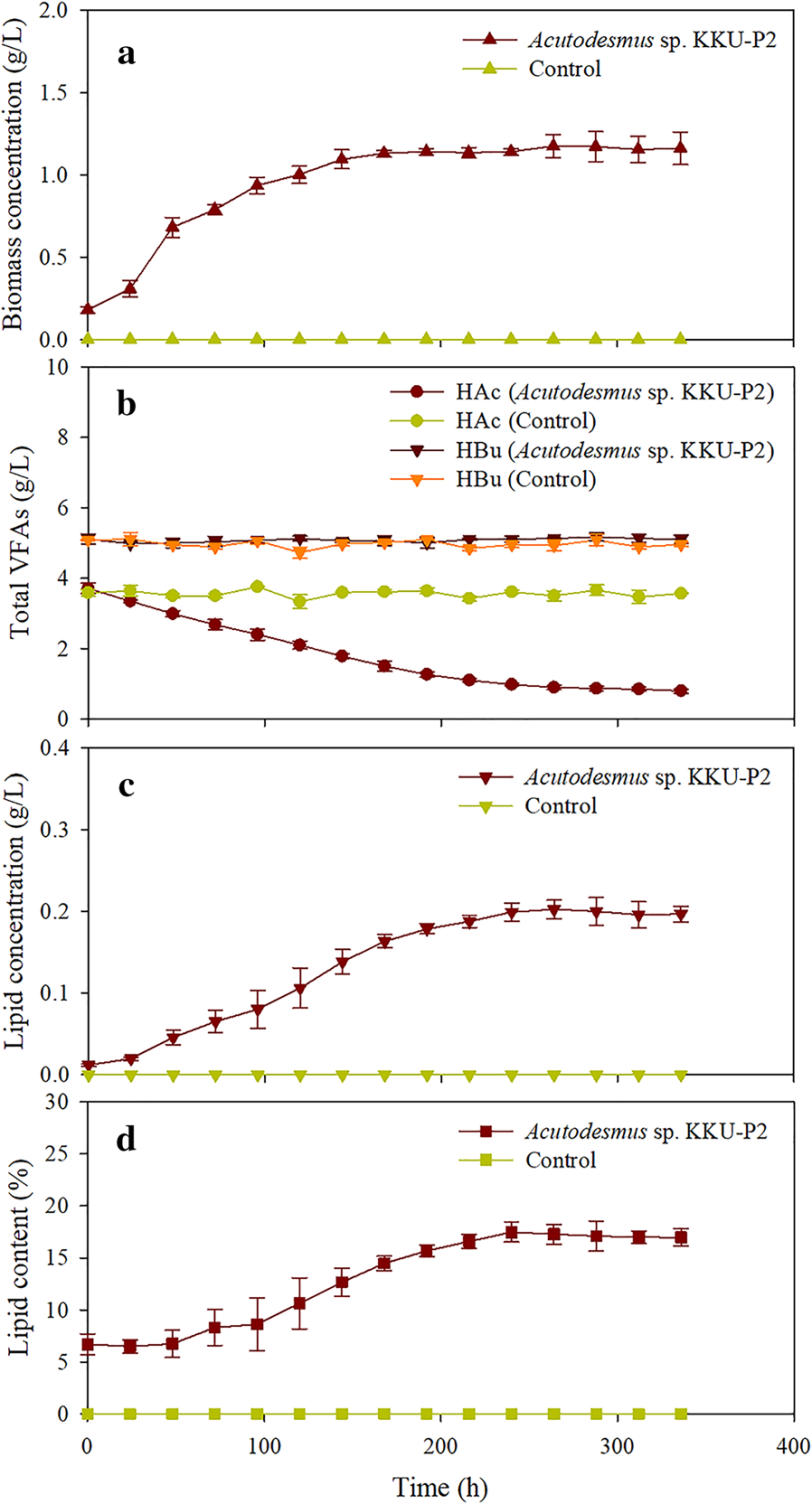
Time-course profiles of cell growth and lipid production. **a** Biomass concentration, **b** total VFAs concentration, **c** lipid concentration, **d** lipid content. The lipids were produced by fermenting hydrogenic effluent using *Acutodesmus* sp. KKU-P2 at 30 °C and 150 rpm under a light intensity of 3000 lx for 14 days

The hydrogenic effluent from the optimal proportions of feedstock was further used to produce lipid by *Acutodesmus* sp. KKU-P2. The strain KKU-P2 gradually grew and achieved a maximum biomass concentration of 1.17 g/L (Fig. 5a), indicating that it could use VFAs in hydrogenic effluent for their growth. The acetic acid concentration decreased significantly over time, whereas butyric acid remained constant until the end of fermentation (Fig. 5b). The results suggested that strain KKU-P2 preferred acetic acid as the carbon source over other acids such as butyric acid. In addition, acetic acid has a two-carbon atom, making it easy to consume by microalgae for biomass and lipid production in this study. This finding was consistent with that of Ren et al. [30], who reported that dark fermentative effluent with acetate as the main SMPs can be used to cultivate microalgal *Scenedesmus* sp. R-16 for lipid production.

The pattern of lipid concentration and content was similar to that of the biomass concentration profile. Lipid concentration steadily increased to the maximum value of 0.20 g/L at 264 h (Fig. 5c) with the highest lipid content of 17.3% (w/w) (Fig. 5d). At this time, the COD was virtually eliminated with a removal percentage of 20%. The fatty acids found in *Acutodesmus* sp. were oleic (C18:1), palmitic (C16:0), and linoleic acids (C18:2). These results were supported by the previous report of Grama et al. [31], who found that oleic, palmitic and linoleic acids were the significant fatty acid fraction found in *Acutodesmus obliquus*. Microalgae growth and lipid production are influenced by many factors, such as substrate concentration, nutrient content, initial substrate to initial biomass concentration ratio (S/X ratio), light, pH, and temperature [14]. Hence, the optimization of these factors can enhance the lipid content and efficiency of COD removal.

### Analysis of composting properties of solid residues at the end of hydrogen fermentation and the remaining acidified slurries from methane fermentation

The solid residues after the hydrogen fermentation process and the remaining acidified slurries from the methane fermentation process under the optimum proportions of feedstock were analyzed for the composting properties. Results revealed that the chemical properties of the solid residues at the end of hydrogen fermentation passed the compost standard (Table 5). The C/N ratio of 20.22 of the remaining acidified slurries from methane fermentation was slightly higher than the standard of compost (< 20) [32]. Therefore, solid residues can be directly used as soil supplements without being composted.

**Table 5:** Comparison of standard compost with chemical properties of solid residues after the hydrogen fermentation process and the remaining acidified slurries from the methane fermentation process

Characteristic	Criteria	Solid residues from hydrogen production	Remaining acidified slurries from methane production
Electrical conductivity (dS/m)	≤ 6	3.95	5.32
Organic matter (%w/w)	≥ 30	88.92	89.27
Organic carbon (%w/w)	–	51.57	51.78
C/N ratio	≤ 20	12.58	20.22
Germination index (%w/w)	≥ 80	98	87
Total N (%w/w)	≥ 1.0	4.10	2.56
Total P_2_O_5_ (%w/w)	≥ 0.5	2.41	1.89
Total K_2_O (%w/w)	≥ 0.5	1.94	1.28

### The COD flow and market price from a co-digestion of hydrolysate of *Chlorella* sp. biomass with hydrolysate of sugarcane leaf and anaerobic sludge

**Fig. 6:**
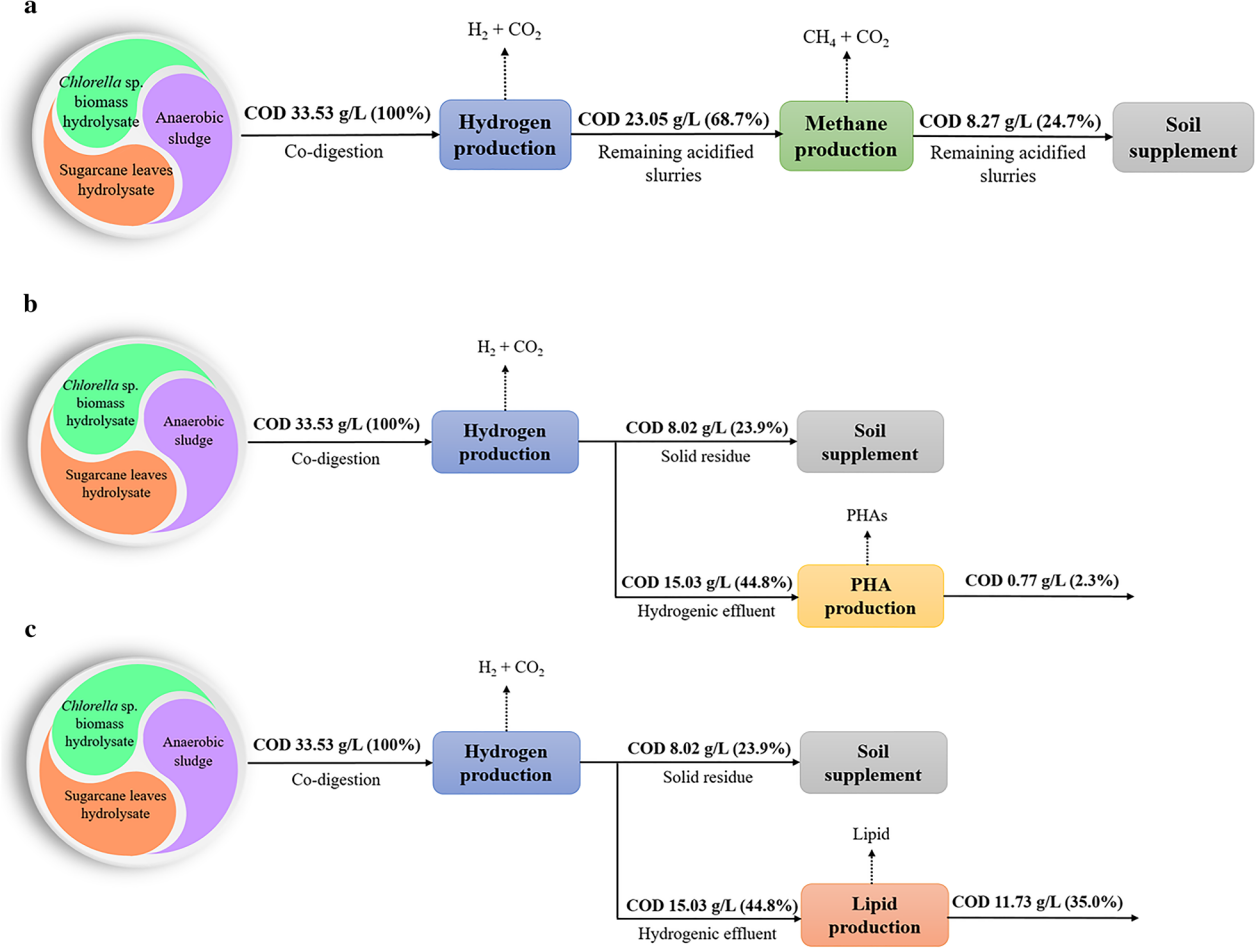
The COD flow and mass balance analysis of organic matter (COD) throughout the different biorefinery schemes. **a** Two-stage hydrogen and methane production; **b** hydrogen and PHA synthesis; **c** hydrogen and lipid synthesis

The efficiency of organic use and recovery in two-stage hydrogen and methane fermentation (24.7%) was higher than that of hydrogen fermentation alone (68.7%) (Fig. 6a). The results suggest that the two-stage hydrogen and methane production process enhanced the organic recovery, resulting in high energy attained. In addition, the remaining acidified slurries from the methane production process showed properties that could be used as soil supplements.

Figure 6b and c demonstrates other biorefinery schemes in which the hydrogenic effluent was converted to produce PHAs (Fig. 6b) and lipids (Fig. 6c). It was found that 97% of organic waste was utilized to produce hydrogen and PHAs (Fig. 6b), whereas only 65% of the organic waste was converted into hydrogen and lipid (Fig. 6c). Subsequently, the solid residues show the properties of the compost. Therefore, the application of the integrated circular biorefinery concept by producing multi-products was shown to be more viable than a single process, enhanced the COD reduction, and resulted in a complete utilization of the biomass.

The market prices for hydrogen, methane, PHAs, and lipid (linoleic acid) were estimated. The use of hydrolysates of *Chlorella* sp. biomass and sugarcane leaf, and anaerobic sludge to produce only hydrogen resulted in a gain of approximately 6.09 US$/kg; in contrast, the production of only methane resulted in a market price gain of 0.24 US$/kg [33] (Additional file 1: Fig. S2). Hence, the integration of two-stage hydrogen and methane results in a market price gain of 6.33 US$/kg. VFAs with a market price of 1.0 US$/kg can be conveniently used as a precursor for PHA and lipid (linoleic acid) synthesis. The market products of PHA and linoleic acid synthesis can have a value of up to 6.09 [33] and 4.0 [34] US$/kg, respectively. Therefore, the integration of hydrogen production with PHAs or lipid synthesis increases the net market price of 12.18 and 10.09 US$/kg, respectively. To commercialize these processes, it is essential to recognize the bottlenecks of processes, such as operational costs, extraction performance, and harvest.

## Conclusions

Biohydrogen production followed by methane, PHA and lipid production is a successful integrated circular biorefinery platform to efficiently utilize the hydrolysates of *Chlorella* sp. biomass and sugarcane leaf. The potential use of the solid residues at the end of hydrogen fermentation and the remaining acidified slurries from methane production as soil supplements demonstrates the zero waste concept. The COD flow clearly indicated that integrated circular biorefinery concept to produce value-added multi-products is more viable than a single process. The approach we have demonstrated in this study provides a foundation for the maximum use of feedstock, resulting in zero waste.

## Materials and methods

### Inoculum preparation

Anaerobic microorganisms contained in anaerobic sludge were used as the inoculum for hydrogen production. They were kindly provided by Kaensiri Starch Co., Ltd. Phra Yuen, Khon Kaen, Thailand. The sludge was heat-treated at 105 °C for 3 h in a hot air oven to inactivate methane-producing bacteria. The sludge was then activated and enriched in a modified basic anaerobic (BA) medium [35] with 5 g/L of glucose as a carbon source in a glass bottle of 400 mL working volume. The initial pH was adjusted to 6.0, using either 5 M NaOH or 5 M HCl. Rubber stoppers and aluminum caps were used to seal the bottles. Anaerobic conditions inside the glass bottle were created by purging with nitrogen gas for 10 min. The culture was incubated at room temperature (30 ± 2 °C) and 150 rpm for 24 h on an orbital shaker. The culture was then transferred to a new BA medium (subculture) and incubated for 24 h. After the enrichment culture was complete, the culture was centrifuged at 5974 g for 10 min to harvest the sludge.

For methane production, anaerobic microorganisms contained in anaerobic sludge were used as the inoculum without heat treatment and were cultivated in the medium, as previously described. The initial pH of the BA was adjusted to 7.0, using 5 M NaOH and 5 M HCl. The culture was incubated at room temperature (30 ± 2 °C) and 150 rpm for 24 h for 3 days before use as a methane producer.

*C. necator* (former name: *Alcaligenes eutrophus* TISTR 1335), purchased from the Thailand Institute of Scientific and Technological Research (TISTR), Thailand, was used as the inoculum for PHA production. It was grown in nutrient broth (NB) using 20 g/L glucose as the carbon source. The culture was incubated at 30 °C and 200 rpm for 18 h. The cells were harvested by centrifugation at 3,500 g 4 °C for 15 min, and re-suspended in the hydrogenic effluent before use.

*Acutodesmus* sp. KKU-P2 (GenBank Accession No. MW555785), kindly provided by Dr. Pensri Plangklang, was used as an inoculum for lipid production. It was isolated from a freshwater fish farming area, Nakhon Ratchasima, Thailand, using a modified Bold’s basal 3 N medium (pH 7.0) with 4.4 mM NaNO_3_ as a nitrogen source and 30% CO_2_ in the air as a carbon source. The inoculum was cultivated in Bold's basal medium [36] with 10 g/L glucose as the carbon source. The culture was incubated in an orbital incubator shaker at 30 °C and 150 rpm under a light intensity of 3,000 lx for 4 days. The cells were then centrifuged at 3500 g 4 °C for 15 min to harvest cells for use in lipid production.

### Feedstock preparation

*Chlorella* sp. biomass was purchased as a dry powder from Yantai Hearol Biotechnology Co., Ltd. Chengmai, Hainan, China. It was stored at − 20 °C in zipper storage bags until use. Before use, the samples were pretreated using an enzymatic pretreatment method. Briefly, 2.6 g *of Chlorella* sp. biomass was added to glass bottles containing 50 mL of distilled water. Mixed enzymes, including cellulase (Cellic^®^ CTec2), glucoamylase (Dextrozyme^®^ GA), and alpha-amylase (Termamyl^®^ SC) from Novozyme, Denmark, were applied to the glass bottle in the appropriate proportions, and the conditions were set according to Giang et al. [37].

Sugarcane leaves were collected from a local sugarcane field in Khon Kaen, Thailand. The sugarcane leaves were milled and sieved through a 0.5-mm screen. Before use, the milled sugarcane leaves was pretreated with alkaline pretreatment followed by enzymatic hydrolysis. Sugarcane leaves (10 g) were pretreated with 100 mL of NaOH (1:10 (w/v)) at various NaOH concentrations (0.5, 1, 1.5, 2, 2.5, and 3% (w/v)). Pretreatment was performed in an autoclave at 121 °C for 60 min. After alkali pretreatment, the solid fraction was filtered through a muslin cloth, rinsed with tap water until pH 7–8, and then dried at 80 °C for 24 h in a hot air oven. Subsequently, 2.5 g of pretreated sugarcane leaves were hydrolyzed with Cellic^®^ CTec2 (Novozyme) at a 35 FPU/g-sugarcane leaves in 50 mL of 0.05 M sodium citrate buffer (pH 5.0) [2] and incubated in a shaking water bath at 50 °C and 150 rpm for 72 h. The TRS concentrations of sugarcane leaf hydrolysate were analyzed using the 3,5-dinitrosalicylic acid (DNS) method. The conditions that gave the highest TRS concentration were further used to optimize the enzyme loadings of 5, 15, 25, 35, 45, 55, and 65 FPU/g-sugarcane leaves. The enzymatic hydrolysis process was conducted as described previously. Subsequently, TRS concentrations in sugarcane leaf hydrolysates were analyzed.

### Statistical design for biohydrogen production

A D-optimal mixture design was applied to determine the optimum proportions of feedstock for hydrogen production. Based on the total initial substrate concentration of 20 g-VS/L, the proportions of *Chlorella* sp. biomass hydrolysate (X_1_), sugarcane leaf hydrolysate (X_2_), and anaerobic sludge (X_3_) were varied according to the design (Table 2) using Design-Expert software (Demo Version 7.0, Stat-Ease Inc., Minneapolis, MN, USA). The hydrogen production potential (P_s_) was used as a response (Table 2). The cubic model describes the relationship between the independent variables, and the response variable is shown in Eq. (2):

2 $$Y = \beta_{0} + \sum \beta_{i} {\rm{X}}_{i} + \sum \beta_{ij} {\rm{X}}_{i} {\rm{X}}_{j} + \sum \beta_{ijk} {\rm{X}}_{i} {\rm{X}}_{j} {\rm{X}}_{k} + \sum \delta_{ij} {\rm{X}}_{i} {\rm{X}}_{j} \left( {{\rm{X}}_{i} - {\rm{X}}_{j} } \right),$$

where Y is the predicted response, *X*_*i*_, *X*_*j*_, and *X*_*k*_ are the independent variables, *β*_0_ is a constant, *β*_*i*_ is the coefficient of individual factors, *β*_*ij*_ and *δ*_*ij*_ are the coefficients of two interacting factors, and *β*_*ijk*_ is the coefficient of the three interacting factors.

### Two-stage hydrogen and methane production

Batch hydrogen fermentation was carried out in 120-mL serum bottles with a working volume of 70 mL. The feedstock was then added to the bottles, as shown in Table 1. The bottles were then filled with a modified BA medium [35] and the working volume was adjusted to 70 mL using distilled water. The initial pH was adjusted to 6.0, using either 5 M NaOH or 5 M HCl. The bottles were sealed with rubber stoppers and aluminum caps. Subsequently, nitrogen gas was flushed into the headspace for 10 min to create anaerobic conditions. All bottles were incubated on an orbital shaker at 30 °C and 150 rpm. All experimental runs were performed in triplicate. During fermentation, the biogas volume was measured using the wetted glass syringe method [38]. The fermentation broth was collected to analyze the concentration of VFAs at the end of fermentation.

Hydrogenic effluent in each experimental run were further used as the substrate for methane production in the second stage. Methane batch fermentation was conducted by adding 30 mL of hydrogenic effluent and 10 g-VS/L of anaerobic sludge into a 60-mL serum bottle. The pH was adjusted to 7.0, using 5 M NaOH. All bottles were sealed and flushed with nitrogen gas again to create anaerobic conditions and incubated under the same conditions as mentioned above. The biogas volume was measured as previously described.

### PHAs and lipid production

Hydrogenic effluent from the optimum proportions of feedstock that gave the highest hydrogen production was used as the substrate for PHA and lipid production. The effluent was centrifuged at 5,974 g for 10 min to remove all suspended solids, and only the supernatants were collected. PHA fermentation was performed in 500-mL Erlenmeyer flasks containing 200 mL of hydrogenic effluent (supernatants), 30% (v/v) of *C. necator* (initial cell concentration 10^7^ CFU/mL), and an initial pH of 7.0. The flasks were incubated in an orbital incubator shaker at 30 °C and 200 rpm for 12 days. Samples were taken during fermentation to analyze the concentrations of sugar residues, VFAs, biomass, PHAs, and COD. The control experiment was performed in the same manner, but without inoculums.

Lipid production was performed in 500-mL Erlenmeyer flasks containing 200 mL of sterile supernatant with 0.75 g/L of NaNO_3_ as a nitrogen source and 0.2 g-DCW/L of *Acutodesmus* sp. KKU-P2 as inoculum. The initial pH was adjusted to 7 using a 5 M NaOH solution. The flasks were incubated at 30 °C and 150 rpm under a light intensity of 3000 lx for 14 days. The control test was conducted in the same manner without KKU-P2. During fermentation, the fermentation broth was collected to analyze the biomass and lipid concentrations, lipid content, and VFAs concentration.

### The potential use of solid residue and the remaining acidified slurries from two-stage hydrogen and methane production process as soil supplements

The solid residues after hydrogen production and the remaining acidified slurries or mixtures from the methane production process under the optimum proportions were collected to analyze the composting properties, including electrical conductivity, organic matter, organic carbon, C/N ratio, germination index, total nitrogen (N), total phosphorus pentoxide (P_2_O_5_), and total potassium oxide (K_2_O). The analytical values were compared with standard compost values to assess their compost properties.

### Analytical methods

The CHNS/O content of hydrolysates of *Chlorella* sp. biomass and sugarcane leaf and anaerobic sludge was analyzed using a CHNS/O analyzer (Flash 2000, Thermo-Scientific, Italy) at the Scientific Equipment Center, Prince of Songkla University, Thailand. Total solids (TS), VS, moisture, and ash content were determined using standard methods [39]. The pH and COD were measured using a pH meter (pH-500, Queen, USA) and a SpectroQuant^®^ COD cell test kit (Merck, Germany), respectively. Cellulose, hemicellulose, and lignin contents were analyzed following the method described by Goering and Van Soest [40]. The total sugar concentration was measured using the phenol–sulfuric acid method [41], and the TRS concentration was determined using the DNS method [42] with glucose as a standard. The TRS yield of sugarcane leaf hydrolysate was calculated by subtracting the TRS concentrations of the hydrolysate by the TRS concentration of the enzyme. The lipid content of microalgae was determined using the colorimetric sulfo-phospho-vanillin (SPV) method [43]. The electrical conductivity was determined using a conductivity/DO meter (CyberScan CD650, Eutech, Singapore). The organic carbon and organic matter in the soil supplement samples were measured using the Walkley–Black method [44]. The germination index of the soil supplement samples was analyzed according to the method described by Czabator [45]. Total nitrogen (TN) was determined using the Kjeldahl method [46]. Total phosphorus pentoxide (P_2_O_5_) and total potassium oxide (K_2_O) were analyzed as described by Cock et al. [47].

The biomass concentration in terms of DCW (g/L) was measured using a hot air oven at 80 °C for 24 h. PHAs were extracted using the sodium hypochlorite digestion method described by Poomipuk et al. [48]. Briefly, 5-mL samples were centrifuged at 5,204 g for 10 min and leached with 1 mL distilled water to harvest the cells. The cell solutions were suspended in 2 ml of 6% sodium hypochlorite with 2 ml of chloroform and then incubated at 37 °C and 250 rpm for 2.5 h. PHA granules were collected by centrifugation at 774 g for 20 min and filtered through a 0.45 μm nylon membrane filter. It was then precipitated with 5 mL of methanol and refrigerated at 4 °C for 24 h. PHA pellets were filtered using Whatman filter paper, evaporated, and dried in a hot air oven at 80 °C for 24 h. The weight of the PHAs was measured using a 4-digit weighing balance. The PHA content (% w/w) and residual cell concentration (g/L) were calculated [48].

VFA concentration was determined using high-performance liquid chromatography (HPLC) (Shimadzu LC- 20AD, Tokyo, Japan) following the method of Sitthikitpanya et al. [49]. Hydrogen and methane production were analyzed using gas chromatography (GC) (Shimadzu GC-2014, Tokyo, Japan) [49]. The hydrogen and methane volumes were calculated using the mass balance equation presented by Zheng and Yu [50]. The modified Gompertz equation predicted kinetic parameter values for hydrogen and methane production [51]. HY, MY, and EY were calculated as described by Reungsang et al. [52].

The synergistic and antagonistic effects of co-digestion of *Chlorella* sp. biomass hydrolysate, sugarcane leaf hydrolysate, and anaerobic sludge were calculated according to Wadjeam and Reungsang [6].

## Supplementary Information

**Additional file 1: Table S1**. Compositions of *Chlorella* sp. biomass hydrolysate, sugarcane leaf hydrolysate, and anaerobic sludge.

**Additional file 2: Table S2**. ANOVA for the cubic model regression representing hydrogen production potential in mixture design.

**Additional file 3: Figure S1**. The mixture design plots for hydrogen production potential from the co-digestion process. (a) 3D surface, (b) contour.

**Additional file 4: Figure S2**. The hydrogen, methane, PHAs, and lipid (linoleic acid) production and the market prices of various products.

## Data Availability

The datasets used and/or analyzed during the current study are available from the corresponding author on reasonable request.

## Abbreviations

C/N: Carbon to nitrogen
VFAs: Volatile fatty acids
VS: Volatile solid
PHAs: Polyhydroxyalkanoates
COD: Chemical oxygen demand
HY: Hydrogen yield
MY: Methane yield
EY: Energy yield
HRT: Hydraulic retention time
EFB: Empty fruit bunches
POME: Palm oil mill effluent
SCW: Second cheese whey
CCWP: Concentrated cheese whey permeate
VSS: Volatile suspended solids
DCW: Dry cell weight
H_2_SO_4_: Sulfuric acid
BA: Basic anaerobic
NaOH: Sodium hydroxide
HCl: Hydrochloric acid
TISTR: Thailand Institute of Scientific and Technological Research
NB: Nutrient broth
FPU: Filter paper unit
TRS: Total reducing sugar
DNS: 3,5-Dinitrosalicylic acid
P_s_: Hydrogen production potential
NaNO_3_: Sodium nitrate
P_2_O_5_: Phosphorus pentoxide
K_2_O: Potassium oxide
CHNS/O: Carbon–hydrogen–nitrogen–sulfur–oxygen
TS: Total solids
SPV: Sulfo-phospho-vanillin
DO: Dissolved oxygen
HPLC: High-performance liquid chromatography
GC: Gas chromatography
LCC: Lignin–carbohydrate complex
R^2^: Coefficient of determination
3D: Three-dimensional
SMPs: Soluble metabolite products
FTIR: Fourier transform infrared spectrometer
PHB: Polyhydroxybutyrate
S/X: Initial biomass concentration
X_1_: *Chlorella* sp. biomass hydrolysate
X_2_: Sugarcane leaf hydrolysate
X_3_: Anaerobic sludge
