# Supplementary material for: Co-generation of biohydrogen and biochemicals from co-digestion of Chlorella sp. biomass hydrolysate with sugarcane leaf hydrolysate in an integrated circular biorefinery concept
Source: Biotechnol Biofuels. 2021 Oct 1;14:197. doi: 10.1186/s13068-021-02041-6 (PMC8487135; doi:10.1186/s13068-021-02041-6)
Supplement: Supplementary file 1 — Additional file 1: Table S1. Compositions of Chlorella sp. biomass hydrolysate, sugarcane leaf hydrolysate, and anaerobic sludge. [file 13068_2021_2041_MOESM1_ESM.pdf]

Table S1 Compositions of *Chlorella* sp. biomass hydrolysate, sugarcane leaf hydrolysate, and anaerobic sludge.

**Parameters**	***Chlorella* sp. biomass hydrolysate**	**Sugarcane leaf hydrolysate**	**Anaerobic sludge**
Total solid (% w/v)	6.72	5.04	13.18
Volatile solid (% w/v)	6.34	4.43	7.30
Moisture (% w/v)	93.28	94.96	86.82
Ash (% w/v)	0.38	0.60	5.88
Nitrogen (% w/w)	6.75	0.38	4.63
Carbon (% w/w)	43.04	37.35	30.92
Hydrogen (% w/w)	6.79	6.42	4.54
Sulfur (% w/w)	0.56	0.02	0.63
Oxygen (% w/w)	35.70	50.68	19.16
C/N ratio	6.38	99.08	6.68
