# Supplementary material for: Co-generation of biohydrogen and biochemicals from co-digestion of Chlorella sp. biomass hydrolysate with sugarcane leaf hydrolysate in an integrated circular biorefinery concept
Source: Biotechnol Biofuels. 2021 Oct 1;14:197. doi: 10.1186/s13068-021-02041-6 (PMC8487135; doi:10.1186/s13068-021-02041-6)
Supplement: Supplementary file 2 — Additional file 2: Table S2. ANOVA for the cubic model regression representing hydrogen production potential in mixture design. [file 13068_2021_2041_MOESM2_ESM.pdf]

Table S2 ANOVA for the cubic model regression representing hydrogen production potential in mixture design.

Source	Sum of Squares	df	Mean Square	F-value	p-value Prob > F
Model	18468521.84	9	2052057.98	96.47	< 0.0001
Linear Mixture	10300007.21	2	5150003.60	242.12	< 0.0001
X_1X_2	175654.32	1	175654.32	8.26	0.0184
X_1X_3	360560.97	1	360560.97	16.95	0.0026
X_2X_3	2384583.57	1	2384583.57	112.11	< 0.0001
$X_1X_2X_3$	1044491.88	1	1044491.88	49.10	< 0.0001
$X_1X_2 (X_1- X_2)$	51065.23	1	51065.23	2.40	0.1557
$X_1X_3 (X_1- X_3)$	123057.56	1	123057.56	5.79	0.0396
$X_2X_3 (X_2- X_3)$	517832.99	1	517832.99	24.34	0.0008
Residual	191435.99	9	21270.67		
Lack of Fit	83027.11	2	41513.56	2.68	0.1367
Pure Error	108408.88	7	15486.98		
Cor Total	18659957.83	18			
R^2 = 0.9897; Adj R^2 = 0.9795					
