# Supplementary material for: Co-generation of biohydrogen and biochemicals from co-digestion of Chlorella sp. biomass hydrolysate with sugarcane leaf hydrolysate in an integrated circular biorefinery concept
Source: Biotechnol Biofuels. 2021 Oct 1;14:197. doi: 10.1186/s13068-021-02041-6 (PMC8487135; doi:10.1186/s13068-021-02041-6)
Supplement: Supplementary file 4 — Additional file 4: Figure S2. The hydrogen, methane, PHAs, and lipid (linoleic acid) production and the market prices of various products. [file 13068_2021_2041_MOESM4_ESM.pdf]

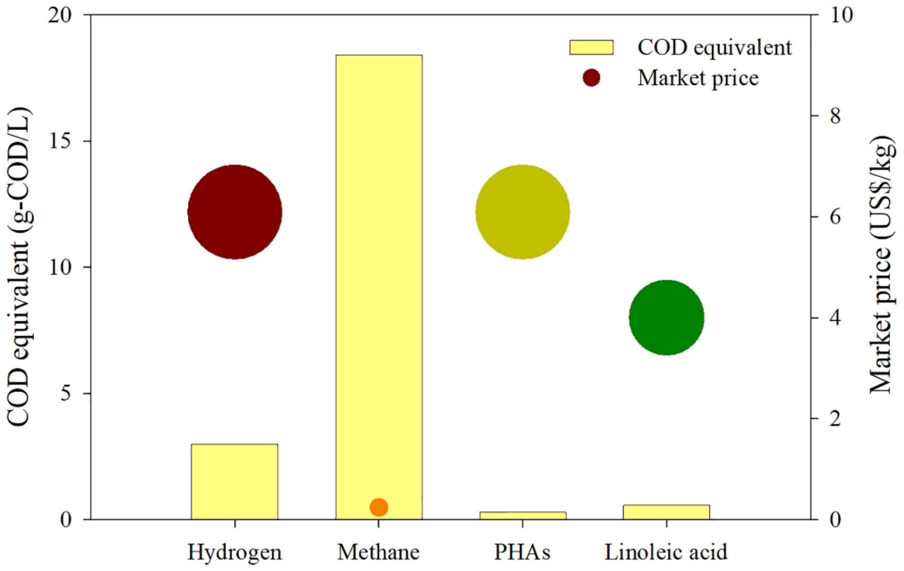

Fig. S2 The hydrogen, methane, PHAs, and lipid (linoleic acid) production and the market prices of various products.
