# Supplementary material for: Co-generation of biohydrogen and biochemicals from co-digestion of Chlorella sp. biomass hydrolysate with sugarcane leaf hydrolysate in an integrated circular biorefinery concept
Source: Biotechnol Biofuels. 2021 Oct 1;14:197. doi: 10.1186/s13068-021-02041-6 (PMC8487135; doi:10.1186/s13068-021-02041-6)
Supplement: Supplementary file 3 — Additional file 3: Figure S1. The mixture design plots for hydrogen production potential from the co-digestion process. (a) 3D surface, (b) contour. [file 13068_2021_2041_MOESM3_ESM.pdf]

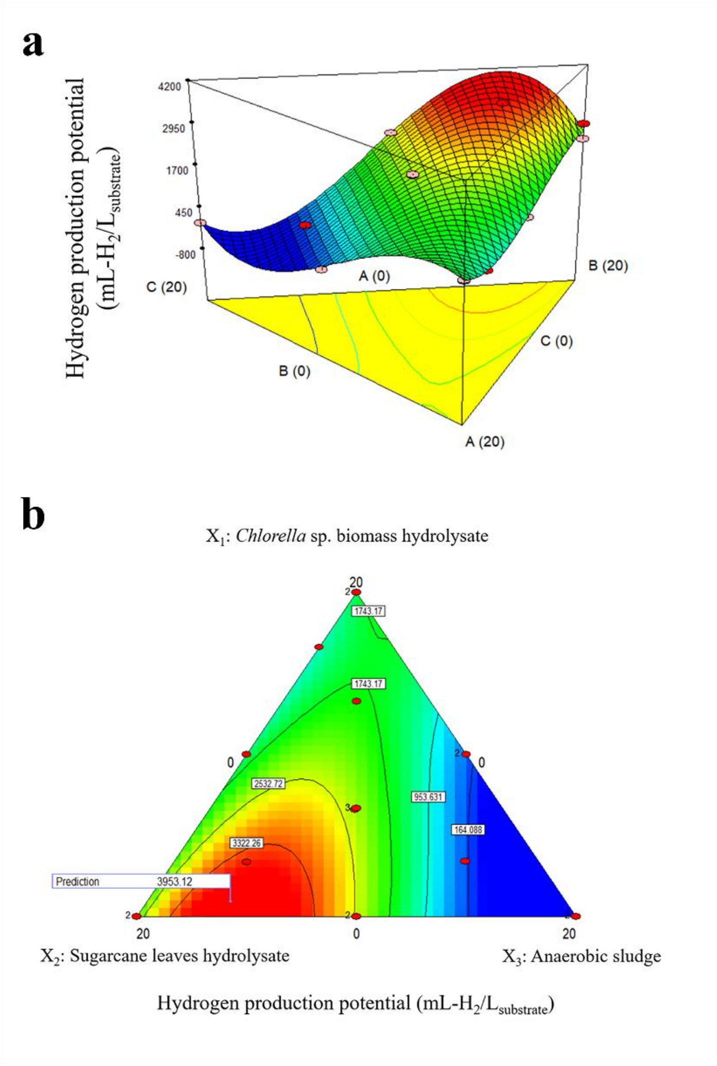

Fig. S1 The mixture design plots for hydrogen production potential from the co-digestion process.

(a) 3D surface, (b) contour.
